# The effect of motor skills and imagery application on psychomotor development in children

**DOI:** 10.3389/fpsyg.2025.1682612

**Published:** 2026-01-28

**Authors:** Ozan Yilmaz, Betul Bayazit

**Affiliations:** 1 Department of Recreation, Faculty of Sport Science, Istanbul Yeni Yuzyil University, İstanbul, Türkiye; 2 Department of Recreation, Faculty of Sport Science, Kocaeli University, Kocaeli, Türkiye

**Keywords:** motor skill, psychomotor, imagery, experimental research, fine and gross motor skills

## Abstract

This study aimed to investigate the psychomotor development effect of motor skills and imagery application in 13 year-old boys. The research group consisted of 40 male volunteer children who had 13 studied at Kocaeli/İzmit 29 Ekim Secondary School. The research groups were divided into four groups (control, experiment-1, experiment-2, experiment-3) had 13 with a random method as n=10 children per group. A skill track consisting of eight stations, including fine and gross motor skills, was applied as a data collection tool. Motor skills and imagery programs were applied to the experimental groups 2 days a week for 8 weeks. The control group did not participate in imagery and motor skills exercises. The station scores and track finishing time that constitute the results of the pre- and post-tests of the intervention were recorded. Data analysis was performed using SPSS 21.0. According to the normality test results, the Wilcoxon test, Kruskal–Wallis test, paired samples test, and one-way ANOVA test were applied. There were statistically significant differences between the study groups in balance, ball transport with racket, target ball throwing, cross hopping, ball driving, shooting, and over the hurdles practice post-test station score averages (*p*<0.05). There were significant differences between the total score on the track and the post-test averages of the track finish time (*p*<0.05). Imagery application studies are important mental processes in the uptake of motor learning and motor development. This research, which will be a reference for future studies, emphasizes the importance of mental preparation and states that the repetitive physical work method is also effective.

## Introduction

Movement is crucial for children’s physical development. Currently, children’s movement areas are extremely limited (Tascı, 2010). One of the first developments to consider when children are in motion is motor development. Motor Development involves the development of movement tasks throughout a person’s life. This evolution reflects the constant change in motor behavior resulting from the interaction between an individual’s biology and environmental conditions (Gallahue et al., 2014). Psychomotor development refers to the acquisition of mobility (Akandere, 2003) that occurs through the development of the brain and spinal cord, along with physical growth and maturation of the organism. Based on this information, the movement or training of motor skills can be expressed as having a significant impact on the development of psychomotor skills.

At the same time, it is necessary to fulfill the application by making informed, choosing appropriate technical choices, and utilizing effective methods in order to achieve successful results in sports skills. Development work can make significant contributions to the development of athletes’ ability to make the right decisions and apply their skills effectively (Konter, 1999).

The use of imagery for performance enhancement in sports can be addressed in various ways. It has been argued that physical and mental study disciplines can only be more effective in achieving motor skills than physical work, and that the development of physical skills may help to learn and improve motor skills (Feltz and Landers, 1983).

There are no definitive consequences of how much it helps to study the learning of completely new skills. However, since these skills are seen several times in the beginning and their design is formed in the brain, mental training is thought to be beneficial (Konter, 1999).

Studies indicate that motor skill interventions are effective for the acquisition of fundamental movement skills, particularly in children aged 3–12 years (Logan et al., 2017). However, the age of 13 years holds distinct significance as it represents the transitional period from childhood to adolescence. During this stage, significant changes are observed in biological maturation, motor coordination, and visuospatial skills (Ju et al., 2018). However, there is a notable scarcity of studies that focus specifically on the 13-year-old age group as a distinct sample. Most research has generally focused on broader age ranges, such as 9–12 or 12–15 years. In this context, we aimed to investigate the effects of motor skills and imagery application on psychomotor development in children aged 13 years. This study’s novel contribution lies in demonstrating the potential of a more comprehensive and effective educational model for children’s motor learning and performance achieved by combining two different training modalities. It is anticipated that these findings will shed light on the development of new evidence-based training strategies and provide valuable guidance for coaches, physical education teachers, and sports scientists.

## Materials and methods

### Type of research

This research is based on the pre-test/post-test control group design within the experimental method and thus has an experimental design.

### Participants

The research group consisted of 40 voluntary 13-year-old children enrolled in 29 Ekim Secondary Schools in Izmit, Kocaeli, and Türkiye. The following criteria were used for the inclusion of male children in the study:

Received criteria

- 13 years of age
- Not being a licensed athlete
- Absence of an ongoing disability
- No health problems
- Being signed by the guardian of the enlightened consent form and volunteering

Exclusion criteria

- Being a licensed athlete
- Presence of a chronic disease
- Health conditions preventing participation in sports
- Refusal to participate voluntarily

As shown in Table 1, the average height of the control group was 148.70 ± 4.71 cm, and the average body weight was 38.90 ± 7.46 kg. The average height of the motor skill group was 149.90 ± 7.78 cm, and the average body weight was 43.70 ± 13.28 kg. The average height of the imagery group was 145.80 ± 4.77 cm, and the average body weight was 39.10 ± 7.57 kg. The average height of the motor skill and imagery group was 148.80 ± 5.95 cm, and the average body weight was 43.60 ± 10.58 kg. There were no significant differences between the heights and body weights in the study groups (*p* > 0.05).

**Table 1:** Average values of research groups for height and body weight, and one-way ANOVA results.

Variable	Groups	*n*	*X̅*±SS	MIN	MAX	*F*	*p*
Height (cm)	Control group	10	148.70 ± 4.71	143	157	0,871	0,465
	Motor skill group	10	149.90 ± 7.78	140	162		
	Imagery group	10	145.80 ± 4.77	139	155		
	Motor skill + imagery group	10	148.80 ± 5.95	142	160		
Body weight (kg)	Control group	10	38.90 ± 7.46	29	52	0,719	0,547
	Motor skill group	10	43.70 ± 13.28	30	70		
	Imagery group	10	39.10 ± 7.57	30	51		
	Motor skill + Imagery group	10	43.60 ± 10.58	33	66		

### Ethics committee approval

Ethical approval for the study was obtained from Kocaeli University’s non-interventional Clinical Research Ethics Committee on 12.04.2017 with project numbers 2017/67 and 2017/58.

### Procedure

Children’s height and weight were measured in the study. The skill course, which consists of eight stations with fine and gross motor skills, was then applied by the researcher based on the opinions of experts. The station points and track finishing times that comprised the preliminary test results were recorded.

A sample size of 40 participants, consisting of four study groups with 10 participants in each group, was determined based on previous studies with similar experimental designs (Robin et al., 2007; Fontani et al., 2007; Asa et al., 2014; Abraham et al., 2017).

The control group was excluded from the study after 8 weeks.

The experimental-1 (motor skill) group motor skill program was applied for 8 weeks, with a duration of 2 days per week, 60 min per day (Appendix Table A1).

The experiment-2 (imagery) group has been applied to the development program for 8 weeks and 2 days per week for 30 min per day (Appendix Table A2).

The experiment-3 (motor skill and imagery) group was subjected to the motor skill and imagery program for 8 weeks and 2 days per week, for 90 min per day (Appendix Tables A1, A2).

The session durations of the intervention programs were structured based on methodological standards and best practices for each training type, as established in the literature. Accordingly, the 60-min session for the Experiment-1 group, which received only motor skill training, was determined to be the ideal duration for a standard technical training period encompassing warm-up, main work, and cool-down phases (Çakıroğlu and Sökmen, 2012; Maria, 2014; Eskiyecek et al., 2020). The 30-min duration established for the Experiment-2 group, which engaged solely in imagery training, is considered a sufficient and effective timeframe for a holistic mental training session, including relaxation, application of imagery scenarios, and post-session evaluation stages (Guillot et al., 2009). Finally, the 90-min combined session for the Experiment-3 group was designed as a logical integration to allocate adequate time to both the motor skill and imagery components without compromising their effectiveness. This approach is consistent with similar studies that implemented combined protocols (Frank et al., 2014).

Training was conducted by the researcher.

At the end of 8 weeks, the skill track, consisting of eight stations with fine and gross motor skills, was reapplied, and the station scores and track finishing times were recorded, forming the final test results. The practice of measuring, training, and skill testing was conducted at 29 Ekim Secondary School Gym. A skill test was conducted by teachers and researchers from 29 Ekim Secondary School of Physical Education.

### Data collection tool

#### Anthropometric measurements

The size and weight of the children’s anthropometric measurements were recorded at the beginning of the 8 weeks of work.

Height: When the body is upright, the heels are adjacent, the head is in the Frankfort position, and the distance between the point on the head and the wall is measured using a wall scale (Ozer, 2009).

Body weight: When there was a slight garment on the subjects, the electronic weight with bare feet and 0.1 sensitivity was measured by Tanita TBF 300 (Ozer, 2009).

#### Skill test

The skill test used in this study, consisting of eight stations designed to assess fine and gross motor skills, was developed by the researcher. To ensure scientific rigor, the test’s content validity was established through consultation with subject matter experts. Following their feedback, the protocol was finalized and pilot tested on a sample group similar to the study participants to confirm its reliability and standardize the administration procedures. This process ensured that the test was both valid and reliable for data collection.

Station 1st: Balance (3-meter ground balance).

Station 2nd: Ball transport with racket.

Station 3rd: Target ball throwing (3 pieces).

Station 4th: Cross-hopping (Forward, Backward, Right, Left Jump).

Station 5th: Ball driving and shooting.

Station 6th: Ball change.

Station 7th: Over the hurdles (top-bottom).

**Figure 1:**
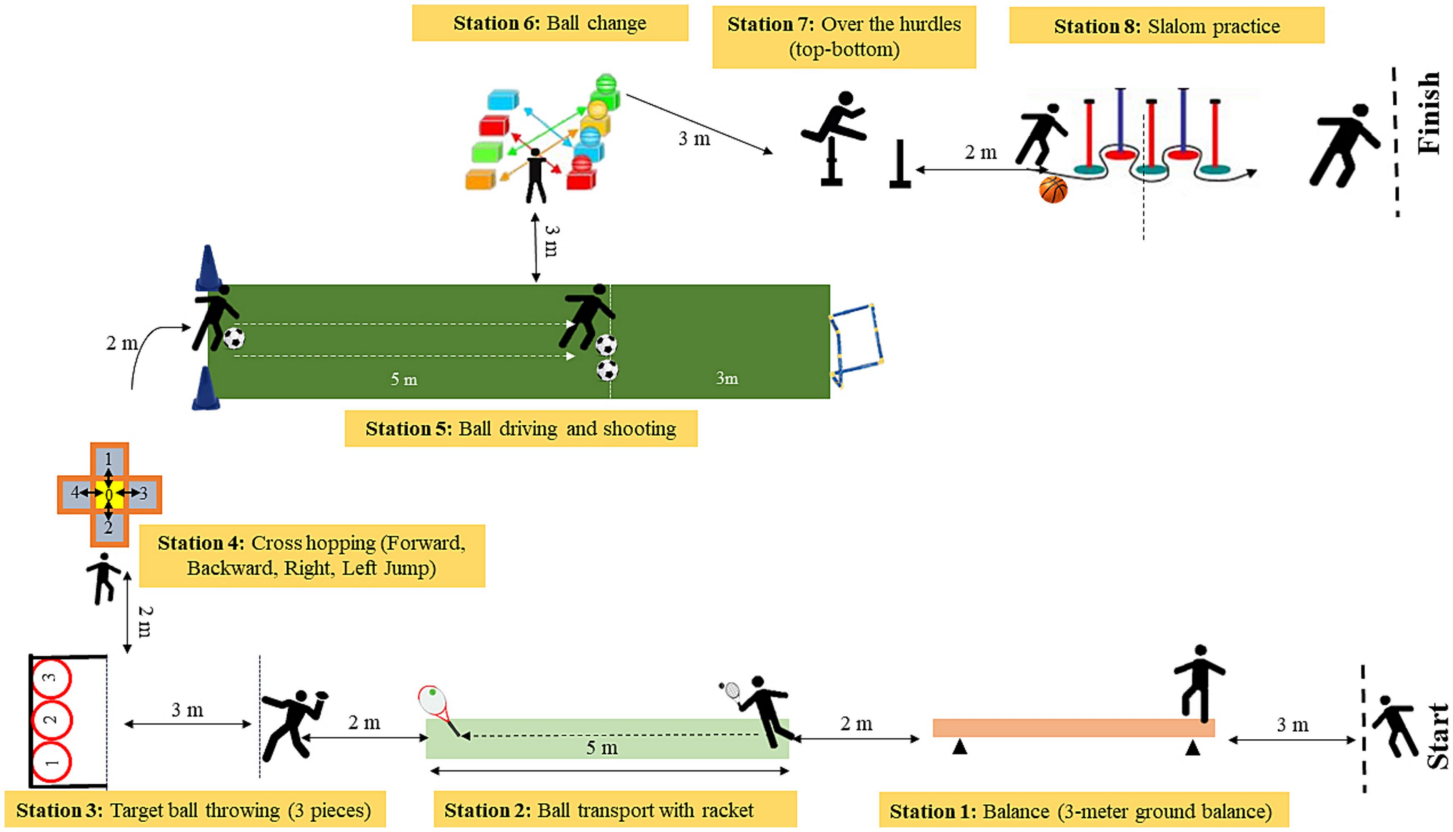
Skill track.

Station 8th: Slalom practice (Figure 1).

A ruler was created for each child to record the station and course end times.

The duration of the course was measured using a Yerlikaya brand wireless photocell chronometer.

### Data analysis

Data analysis was performed using SPSS version 21.0. Descriptive statistics for the data were calculated, and a normality test was performed. According to the Shapiro–Wilk test, Wilcoxon test, and Kruskal–Wallis test were applied for the analysis of station scores that did not show normal distribution in intra- and inter-group analyses. The paired samples test and one-way ANOVA test were applied for the analysis of the track total score and track finishing time, as these demonstrated a normal distribution. To determine the source of the difference between the *post hoc* tests, Tukey’s test was performed. In addition, Cohen’s *d* was calculated based on the means and standard deviations between variables, and the effect size was calculated by considering these results. The data were evaluated at a 95% confidence interval (CI) at a significance level of *p* < 0.05.

## Results

As shown in Table 2, there was no significant difference between the pre-test station score averages of the research groups (*p* > 0.05).

**Table 2:** Pre-test of research groups’ station score averages Kruskal–Wallis test results.

Stations	Groups	*n*	*X̅*±SS	Chi-square	df	*p*
Balance	Control	10	8.40 ± 1.26	2.143	3	0.543
	Motor skill	10	7.50 ± 1.58			
	Imagery	10	7.80 ± 1.54			
	Motor skill + Imagery	10	8.10 ± 1.44			
	Total	40	7.95 ± 1.44			
Ball transport with racket	Control	10	6.60 ± 3.33	0.436	3	0.933
	Motor skill	10	7.00 ± 3.49			
	Imagery	10	7.00 ± 3.49			
	Motor skill + Imagery	10	6.50 ± 2.41			
	Total	40	6.77 ± 3.10			
Target ball throwing	Control	10	4,00 ± 3.94	3.579	3	0.311
	Motor skill	10	1.50 ± 3.37			
	Imagery	10	3.50 ± 3.37			
	Motor skill + Imagery	10	2.50 ± 3.53			
	Total	40	2.87 ± 3.56			
Cross-hopping	Control	10	14.20 ± 1.98	4.245	3	0.236
	Motor skill	10	12.80 ± 2.34			
	Imagery	10	14.40 ± 2.27			
	Motor skill + Imagery	10	12.80 ± 2.34			
	Total	40	13.55 ± 2.28			
Ball driving and shooting	Control	10	13.50 ± 4.11	1.232	3	0.745
	Motor skill	10	15.00 ± 4.71			
	Imagery	10	13.00 ± 5.37			
	Motor skill + Imagery	10	14.50 ± 4.37			
	Total	40	14.00 ± 4.55			
Ball change	Control	10	10.80 ± 2.52	2.281	3	0.516
	Motor skill	10	12.00 ± 0.00			
	Imagery	10	12.00 ± 0.00			
	Motor skill + Imagery	10	10.60 ± 4.42			
	Total	40	11.35 ± 2.53			
Over the hurdles	Control	10	6.40 ± 2.79	1.114	3	0.774
	Motor skill	10	6.40 ± 2.06			
	Imagery	10	6.40 ± 2.06			
	Motor skill + Imagery	10	7.20 ± 1.68			
	Total	40	6.60 ± 2.13			
Slalom practice	Control	10	6.50 ± 3.37	3.023	3	0.388
	Motor skill	10	8.00 ± 3.49			
	Imagery	10	6.00 ± 4.59			
	Motor skill + Imagery	10	8.50 ± 2.41			
	Total	40	7.25 ± 3.57			

*p < 0.05.

As shown in Table 3, there was no significant difference between the total track score of the research groups and the pre-test period of the track finish time (*p* > 0.05).

**Table 3:** One-way ANOVA results of the research groups’ track total score and track finishing time pre-test averages.

Variable	Groups	*X̅*±SS	df	*F*	*p*
Track total score	Control	70.40 ± 09.44	3	0.005	0.999
	Motor skill	70.20 ± 11.97			
	Imagery	70.10 ± 14.23			
	Motor skill + Imagery	70.70 ± 11.02			
	Total	70.35 ± 11.02			
Track finishing time	Control	56:39:48 ± 09:56:55	3	0.053	0.983
	Motor skill	56:36:18 ± 06:48:46			
	Imagery	56:22:00 ± 05:43:42			
	Motor skill + Imagery	57:39:36 ± 08:09:46			
	Total	56:49:25 ± 07:32:00			

As shown in Table 4, there was no significant difference between the ball change of the research groups and the post-test averages of the slalom practice station scores (*p* > 0.05). However, there was a significant difference in the station score between balance, ball transport with a racket, target ball throwing, cross-hopping, ball driving, shooting, and running over hurdles (*p* < 0.05).

**Table 4:** Post-test of the station score averages of research groups Kruskal–Wallis test results.

Stations	Groups	*n*	*X̅*±SS	Chi-square	df	*p*	Difference	Cohen’s *d*
Balance	Control (1)	10	6.30 ± 0.94	18.796	3	0.000*	1–2 1–4 3–4	1.89 4.06 1.47
	Motor skill (2)	10	8.40 ± 1.26					
	Imagery (3)	10	6.90 ± 2.02					
	Motor skill + Imagery (4)	10	9.00 ± 0.00					
	Total	40	7.26 ± 1.65					
Ball transport with racket	Control (1)	10	6.50 ± 2.41	11.471	3	0.009*	1–4 2–4 3–4	2.05 1.09 1.64
	Motor skill (2)	10	8.00 ± 2.58					
	Imagery (3)	10	7.00 ± 2.58					
	Motor skill + Imagery (4)	10	10.00 ± 0.00					
	Total	40	7.87 ± 2.50					
Target ball throwing	Control (1)	10	3.50 ± 3.37	19.202	3	0.000*	1–2 1–4 3–4	2.29 2.83 1.15
	Motor skill (2)	10	11.00 ± 3.16					
	Imagery (3)	10	7.50 ± 4.85					
	Motor skill + Imagery (4)	10	12.00 ± 2.58					
	Total	40	8.50 ± 4.83					
Cross-hopping	Control (1)	10	11.80 ± 2.20	13.075	3	0.004*	1–4 2–4 3–4	1.56 1.10 1.61
	Motor skill (2)	10	13.40 ± 0.96					
	Imagery (3)	10	12.60 ± 1.34					
	Motor skill + Imagery (4)	10	14.40 ± 0.84					
	Total	40	13.05 ± 1.69					
Ball driving and shooting	Control (1)	10	14.00 ± 2.10	17.531	3	0.001*	1–2 1–4 2–3 3–4	1.11 1.99 1.11 1.99
	Motor skill (2)	10	16.50 ± 2.41					
	Imagery (3)	10	14.00 ± 2.10					
	Motor skill + Imagery (4)	10	18.50 ± 2.41					
	Total	40	15.75 ± 2.89					
Ball change	Control (1)	10	11.30 ± 1.49	6.250	3	0.100	–	–
	Motor skill (2)	10	12.00 ± 0.00					
	Imagery (3)	10	12.00 ± 0.00					
	Motor skill + Imagery (4)	10	12.40 ± 1.26					
	Total	40	11.92 ± 1.02					
Over the hurdles	Control (1)	10	5.60 ± 2.06	11.125	3	0.011*	1–2 1–4 3–4	1.17 1.65 1.09
	Motor skill (2)	10	7.60 ± 1.26					
	Imagery (3)	10	6.40 ± 2.06					
	Motor skill + Imagery (4)	10	8.00 ± 0.00					
	Total	40	6.90 ± 1.80					
Slalom practice	Control (1)	10	10.00 ± 0.00	0.000	3	1.000	–	–
	Motor skill (2)	10	10.00 ± 0.00					
	Imagery (3)	10	10.00 ± 0.00					
	Motor skill + Imagery (4)	10	10.00 ± 0.00					
	Total	40	10.00 ± 0.00					

*p < 0.05.

As shown in Table 5, a significant difference was found between the total track score of the research groups and the post-test period of track completion time (*p* < 0.05).

**Table 5:** One-way ANOVA results of the post-test averages of the research groups’ total score and track finishing time.

Variable	Groups	*X̅*±SS	df	*F*	*p*	Difference	Cohen’s *d*
Track total score	Control (1)	69.00 ± 08.11	3	31.540	0.000*	1–2 1–3 1–4 2–3 2–4 3–4	2.55 0.98 4.07 1.65 1.58 3.27
	Motor skill (2)	86.90 ± 05.72					
	Imagery (3)	76.40 ± 06.97					
	Motor skill + Imagery (4)	94.30 ± 03.36					
	Total	81.65 ± 11.52					
Track finishing time	Control (1)	60:00:54 ± 08:37:41	3	5.080	0.005*	1–2 1–4	0.99 1.50
	Motor skill (2)	53:11:00 ± 05:17:44					
	Imagery (3)	53:55:54 ± 03:11:27					
	Motor skill + Imagery (4)	49:52:12 ± 05:18:22					
	Total	54:15:00 ± 06:48:04					

*p < 0.05.

As shown in Table 6, the control group showed a decrease in the track total score averages in the pre- and post-tests. There was an increase in the motor skill, imagery, and motor skill and imagery groups in the pre- and post-tests between the track total score averages. If you need to sort groups according to differences.

**Table 6:** Pre-test of research groups and post-test track total score averages and standard deviation difference values.

Groups	*n*	Track total score	*X̅* ± SS
Control	10	Pre-test	70.40 ± 9.44
		Post-test	69.00 ± 8.11
		Difference	−1.40 ± 8.15
Motor Skill	10	Pre-test	70.20 ± 11.97
		Post-test	86.90 ± 5.72
		Difference	16.70 ± 9.20
Imagery	10	Pre-test	70.10 ± 14.23
		Post-test	76.40 ± 6.97
		Difference	6.30 ± 11.55
Motor skill + Imagery	10	Pre-test	70.70 ± 9.55
		Post-test	94.30 ± 3.36
		Difference	23.60 ± 8.80

Motor skill and imagery group (*X̅*_Difference_ = 23.60 ± 8.80) > Motor skill group (*X̅*_Difference_ = 16.70 ± 09.20) > The imagery group (*X̅*_Difference_ = 06.30 ± 11.55) > The control group (*X̅*_Difference_ = −1.40 ± 08.15).

As shown in Table 7, there was an increase in the control group between the pre- and post-test track averages. The motor skill, imagery, and motor skill and imagery groups showed decreases in the pre- and post-test tracks.

**Table 7:** Pre-test of research groups and post-test track finishing time averages and standard deviation difference values.

Groups	*n*	Track finishing time	*X̅* ± SS
Control	10	Pre-test	56:39:48 ± 09:56:55
		Post-test	60:00:54 ± 08:37:41
		Difference	03:21:06 ± 07:22:24
Motor skill	10	Pre-test	56:36:18 ± 06:48:46
		Post-test	53:11:00 ± 05:17:44
		Difference	−03:25:18 ± 02:31:17
Imagery	10	Pre-test	56:22:00 ± 05:43:42
		Post-test	53:55:54 ± 03:11:27
		Difference	−02:26:06 ± 2:50:25
Motor skill + Imagery	10	Pre-test	57:39:36 ± 08:09:46
		Post-test	49:52:12 ± 05:18:22
		Difference	−07:47:24 ± 03:48:16

If you need to sort groups by differences,

Motor skill and imagery group (*X̅*_Difference_ = −07:47:24 ± 03:48:16) > Motor skill group (*X̅*_Difference_ = −03:25:18 ± 02:31:17) > The imagery group (*X̅*_Difference_ = −02:26:06 ± 02:50:25) > Control group (*X̅*_Difference_ = 03:21:06 ± 07:22:24).

## Discussion

In today’s modern world, humankind is now imprisoned by technology; therefore, areas of motion become very limited. However, movement during a child’s physical development, which guarantees the future, is a distinct element. Movement is important for motor development. Therefore, this study aimed to assess the effectiveness of the application of motor skills and imagery in adolescents.

This study was conducted on a 13-year-old boy weighing 40 kg. The height and body weight of the male children who participated in the study were measured before leaving the groups, and then a skill track consisting of eight stations with fine and gross motor skills prepared by the researcher based on expert opinions was applied. The station scores and track finishing times were recorded to obtain pre-test results.

At the end of the measurements of the groups before the study, the control group’s average height was 148.70 ± 4.71 cm, and the average body weight was 38.90 ± 7.46 kg. The average height of the motor skill group is 149.90 ± 7.78 cm, and the average body weight is 43.70 ± 13.28 kg. The imagery group had an average height of 145.80 ± 4.77 cm, and a mean body weight of 39.10 ± 7.57 kg. The motor skill and imagery group had an average height of 148.80 ± 5.95 cm, and the average body weight was 43.60 ± 10.58 kg.

### In-group pre-test of the control group post-test results

In the findings of the study, there was no significant difference between the control group and the ball transport with racket, target ball throwing, ball driving and shooting, ball change, and the scores at the “over the hurdles” station pre-test and post-test measurement results. However, there was a significant difference between the positive directional pre-test and post-test measurement results in the negative directional and slalom practice station balance and cross-hopping station scores.

In this context, control group balance and cross-hopping station scores can be linked to the occurrence of significant differences in the negative direction, not participating in studies. The significant difference in the slalom practice station is the weighted ability of the other stations, which could lead to better running performance and quickness during this stage.

In this study, there was no significant difference between the total track score of the control group and the track finishing time in the pre- and post-test measurement results.

There was a decrease in the total track score of the pre- and post-test tracks in the control group and an increase in the course of the track. This can be attributed to the fact that the control group did not participate in the study.

### In-group pre-test and motor skill group post-test results

In this study, there was no significant difference between the balance of the motor skill group, ball transport with racket, cross-hopping, ball driving and shooting, ball change, over the hurdles, and the slalom practice station scores pre- and post-test measurement results. However, a significant difference was found between the target ball throwing station scores in the pre- and post-tests.

In this study, there was a significant difference between the total track score of the motor skill group and the track finishing time pre- and post-test measurement results.

In the studies carried out, an increase in the track total score of skill development and the course of the degree of course close to 0, with the development of sportive performance, is seen.

### In-group pre-test of the imagery group post-test results

In this study, there was no significant difference between the pre- and post-test measurement results for balance in the imagery group, ball transport with a racket, cross-hopping, ball driving and shooting, ball change, and over the hurdle station. However, there was a significant difference between the target ball throwing and slalom practice scores before and after the intervention.

There was no significant difference between the total track score of the imagery group and the pre- and post-test measurement results. However, there was a significant difference between the pre- and post-intervention results.

As researcher Plessinger stated, the age group most responsive to imagery was between 11.9 and 13.9 years of age (as cited in Kızıldag, 2007, p. 50). In previous studies, it has been effective in scoring certain skills on the skill level of development efforts while increasing chronometric performance.

### In-group pre-test of the motor skill and imagery group post-test results

In this study, there was no significant difference between the motor skill and the imagery group’s balance, cross-hopping, ball change, hurdles, and slalom practice station scores before and after the intervention. However, there was a significant difference between ball transport with rackets, target ball throwing, ball driving, and shooting station scores in pre- and post-test measurement results.

In this study, there was a significant difference between the motor skill and the imagery group’s track total scores and the track finishing time pre- and post-test measurement results.

As a result of the support of motor skill studies with the study of imagery, this situation increased the score in more stations compared to the group that only did the motor skill work, and the track has increased in total points. In this group, we see that the development of skills is increased, and sportive performance is seen by the approach of the course finishing degrees to 0.

### Pre-test results between research groups

There were no significant differences between the balance of the research groups, ball transport with a racket, target ball throwing, cross-hopping, ball driving and shooting, ball change, over the hurdles, and slalom practice pre-test station scores.

There was no significant difference between the total track score of the research groups and the pre-test period of track finish time.

The children involved in the study showed that they were not involved in such studies, were not in an application for skill development, and were evenly divided into groups.

### Post-test results between research groups

There was no significant difference between the ball change of the research groups and the post-test averages of the slalom practice station scores. However, a significant difference was found between balance, ball transport with rackets, target ball throwing, cross-hopping, ball driving, shooting, and over the hurdles in the post-test of the station score.

There was a significant difference between the total track score of the research groups and the post-test period of track completion time.

When we examine the study in the score, the most developed groups are the motor skill and imagery group (*X̅*_Difference_ = 23.60 ± 8.80), the motor skill group (*X̅*_Difference_ = 16.70 ± 09.20), and the control group (*X̅*_Difference_ = −1.40 ± 06.30). The importance of support for motor skill studies has arisen with the development of this study. Both motor skills and imagery applications can support the problems faced by children in accordance with the desired basic technique.

When we examine the work as a track of finishing time, the most developed groups were determined as follows: The motor skill and imagery group (*X̅*_Difference_ = −07:47:24 ± 03:48:16) > the motor skill group (*X̅*_Difference_ = −03:25:18 ± 02:31:17) > the imagery group (*X̅*_Difference_ = −02:26:06 ± 02:50:25) > the control group (*X̅*_Difference_ = 03:21:06 ± 07:22:24). In addition to score development, it has emerged over time. The support of motor skill studies, along with the development of imagery applications, may have led to better performance by focusing on children’s work.

This significant difference in the total score of the motor skill and imagery group compared to all other groups—the motor skill, imagery, and control groups—and in the time-track finishing of the motor skill and imagery groups was found to be significant compared to the control group. Therefore, motor skills and imagery applications are important elements for motor learning and psychomotor development.

Motor skill group: significant difference in target ball throwing score; imagery group: significant difference in target ball throwing and slalom practice station score; motor skill and imagery group: significant difference in ball transport with rackets, target ball throwing, and ball driving and shooting scores. The motor skill program and imagery application may be effective in the development of manipulative skills.

Previous studies have shown that imagery applications in older age groups have a positive impact on motor skills and performance, similar to existing studies that affect the motor development of imagery applications in younger age groups. There are also studies that have reported this finding.

Elci et al. (2013) in their study on the imagery applications of swimming athletes between the ages of 9 and 13. There was no statistically significant difference between the pre- and post-test scores of the athletes in the four different groups. However, they stated that there was a significant difference between the pre- and post-test skill scores of athletes, regardless of group discrimination. Brouziyne and Molinaro (2005), in their study on golfers, found that the goal of golf for beginners is, in combination with the imagery and skill applications of the approach kick, that the imagery-skill group performs better in the approach stroke than the skill group. In a study on elementary school children between 7 and 12 years of age, Caeyenberghs et al. (2009) stated that there is a strong and positive relationship between developmental practices and engine growth and that age is even more effective with progression. Wilson et al. (2002) studied the development of movement coordination of motor imagery in children (imagery group, perception-motor training group, and control group) and found a significant difference between the groups. They stated that the difference in the imagery and perception-motor training groups improved the coordination level of movement. Uludag et al. (2016) studied the effect of imagery and concentration on dart performance in university students and found no statistically significant difference between the control, imagery, and concentration groups in terms of eye fixation time and focus zone. In addition, in the correlation analyses, the post-test in the imagery group indicated a meaningful relationship between the eye fixation period and post-test focus zones. Smith et al. (2007), in his study on women’s gymnastics, used the techniques of imagery in skill learning. In the study, the participants were divided into four groups: the first group used traditional imagery, the second group used PETTLEP imagery, the third group used only physical training, and the fourth group used general gymnastics stretching. At the end of the study, imagery was found to be more effective than conventional imagery in the PETTLEP model Olsson et al. (2008). In the study of high jump athletes for 6 weeks, the experiment group participated in a total of 72 h of imagery program in addition to routine workouts; according to the difference in terms of bounce height, failed jump, bounce angle, and bar distance variables, the result of the study was found only in terms of bar distance in favor of the experiment group between the two groups. Thus, it was concluded that mental training can help develop skill components. Kulak et al. (2011) conducted a study to determine the effect of relaxation, imagery, and regular breathing exercises on some motor characteristics (dynamic balance, flexibility, speed) in 10-12-year-old football players. They stated that regular mental training, together with physical training, was effective in improving the performance of children’s football players.

It is observed that in the studies described above, children’s imagery applications positively affect the development of motor function, while at the same time, learning to develop at a young age provides better control.

The training of coaches to support technical and tactical applications in study programs can help the correct implementation of sports skills by finding an immediate solution to the complex skills that children will encounter. The difference between adults’ and children’s number of workouts and competition/competition counts is the reason that adults use alternative ways to deal with the difficulties encountered. For children with the advantage of preferring solutions with experience, it is possible to develop these features. Therefore, in practice, children should be able to choose the right solutions at an early age, remain calm without excitement, and support themselves with minimal loss of skill. The systematic implementation of the study of imagery has also contributed to the development of the desired basic technique. During this practice, children are asked to act both quickly and practice the correct technique. Naturally, technical development is considered as a whole with technical training, considering the unapplied state of technical training.

This study, which is associated with experimentation with children, shows observable development in experimental groups. As a result, this work on skill and imagery applied to 13-year-old boys is an important thought process for engine learning and the maximization of engine development.

## Conclusion

This work on motor skills and imagery applications applied to 13-year-old boys is important for maximizing motor learning and development. The level of difficulty faced by children before the elite level of sporty performance can improve their ability to choose and practice the right solution by reducing their level of excitement in situations requiring skills. This study, which will serve as a reference for future studies, noted that repeated physical work methods are also effective when emphasizing the importance of mental preparation. The use of imagery techniques during movement training in children can contribute positively to skill learning.

The following suggestions can be considered:

- The use of imagery techniques during movement training in children can contribute positively to skill learning.
- The participation of imagery studies in children’s annual planning can contribute positively to problem-solving and motor learning at an early age.
- Explaining and applying imagery techniques to professional athletes can contribute positively to athletic performance.
- The importance of mental training can be demonstrated in children using different mental training techniques.
- This study can be applied to girls and different age groups.
- As there are very few studies on children in the literature, it is recommended that new studies be planned for children.

### Limitations and future directions

This study is subject to several limitations. The findings obtained in this study are based on a specific sample group and implementation conditions. The study was conducted with 13-year-old male students attending 29 Ekim Secondary Schools in Izmit, Kocaeli, and Türkiye. Although this situation strengthened the internal validity of the results by maintaining the homogeneous nature of the sample, it precluded the evaluation of comparative findings across different age and gender groups. Furthermore, an eight-week intervention period was chosen. While this duration was planned in accordance with the children’s developmental stages and allowed for the observation of short-term effects, longer-term follow-up studies may be beneficial for determining long-term effects. The eight-station skill course used in this study was employed as a valid and reliable tool to assess motor and psychomotor skills. Nevertheless, the scope of measurement being limited to this specific course constrained a detailed examination of various motor skill components. Finally, the study’s focal point was on physical performance indicators. Although this study did not incorporate cognitive and emotional variables, it offers a significant foundation for the physical manifestations of motor skill and imagery interventions.

In future studies, working with larger samples that include different age and gender groups could increase the generalizability of the obtained results. Additionally, extending the intervention period or comparing motor skills and imagery programs of varying intensities may reveal the enduring effects of these methods. The inclusion of cognitive (e.g., attention and focus), emotional (e.g., motivation and self-confidence), and neurophysiological indicators in motor skill and imagery studies could contribute to a more holistic understanding of children’s developmental processes. Similar studies conducted in different socio-economic or cultural settings would be useful in elucidating the effects of environmental factors on psychomotor development. Moreover, studies employing mixed-method (quantitative-qualitative) approaches could enable a deeper understanding of children’s expferiences and learning processes.

## Data Availability

The raw data supporting the conclusions of this article will be made available by the authors, without undue reservation.

## Appendix

**Table A1:** Motor skills program shortened example.

Week and Day	Program Content
1st week|1 st day	Running with knees|5 × 10 m Running with feet hips (Heeling)|5 × 10 m Walking on the speed ladder|5 reps Keeping one leg on the floor and the other leg pulled up on the knee|10 s × 8 reps Kneeling running on the speed ladder|10 reps One leg bounce on the speed ladder|5× right- 5× left Ball bouncing with racket while walking on the ladder|10 reps
4th week, 7th day	Slalom run 5 slalons 3 meters apart|10 reps Keeping one leg on the floor and the other leg pulled up on the knee|10 s × 8 reps One leg bounce on the speed ladder|5× right- 5× left Ball bouncing with racket while walking on the ladder|10 reps Hulahop (circle) waist turning|10 reps Hulahop (hoop) turning on the wrist|5× right -5× left Slalom run with basketball ball 5 slalons 3 m apart|10 reps
6th week, 12th day	Cross leap into one leg hopper (5)|5 s wait ×8 reps Shot on target medicine ball overhead|10 reps Slalom run 5 slalons 3 m apart|10 reps Keeping one leg on the floor and the other leg pulled up on the knee|10 s ×8 reps Slalom dribbling|5 cones ×8 reps Ball bouncing with racket while walking on the ladder|10 reps Slalom run with basketball ball 3 m apart|5 slalons 10 reps
8th week, 15th day	Obstacle bottom crossing|8 reps Slalom run 5 slalons 3 m apart|10 reps One leg on the ground, the other leg pull up on the knee and balance|10 s ×8 reps One leg bounce on the speed ladder|5× right- 5× left Ball bouncing with racket while walking on the ladder|10 reps Slalom run with basketball ball 5 slalons 3 m apart|10 reps

**Table A2:** Imagine program shortened example.

Week and Day		Program Content
1st week	1st day	Breathing exercise
		Inclusion of children in adaptation study. Study on basic concepts.
	2nd day	Breathing exercise
		Ball, obstacle, slalom, racket, hot, cold, beautiful environment, tree, natural environments exercises
2nd week	3rd day	Breathing exercise
		Imagine the details of the skill track, positive basic moves
	4th day	Breathing exercise
		Imagine the details of the skill track, in an environment they imagine, basic concepts
3rd week	5th day	Breathing exercise and recognition phase of the skill track
		Imagery the points to be considered in the skill track as sections, with commands,
	6th day	Breathing exercise
		Located in the skill track; balance, racket handling, target shot imagery
